# Estrogenic Prenylated Flavonoids in *Sophora flavescens*

**DOI:** 10.3390/genes15020204

**Published:** 2024-02-04

**Authors:** Kentaro Nishi, Ikumi Imamura, Kenichiro Hoashi, Ryoiti Kiyama, Shinji Mitsuiki

**Affiliations:** Faculty of Life Science, Kyushu Sangyo University, Fukuoka 813-8503, Japan; nishi@ip.kyusan-u.ac.jp (K.N.); i.imamura0228@gmail.com (I.I.); k20ll128@st.kyusan-u.ac.jp (K.H.); kiyama.r@ip.kyusan-u.ac.jp (R.K.)

**Keywords:** estrogenic activity, prenylated flavonoid, cell signaling pathway, estrogen response, gene expression

## Abstract

*Sophora flavescens* is a medicinal herb distributed widely in Japan and it has been used to treat various diseases and symptoms. To explore its pharmacological use, we examined the estrogenic activity of four prenylated flavonoids, namely kurarinone, kushenols A and I, and sophoraflavanone G, which are characterized by the lavandulyl group at position 8 of ring A, but have variations in the hydroxyl group at positions 3 (ring C), 5 (ring A) and 4’ (ring B). These prenylated flavonoids were examined via cell proliferation assays using sulforhodamine B, Western blotting, and RT-PCR, corresponding to cell, protein, and transcription assays, respectively, based on estrogen action mechanisms. All the assays employed here found weak but clear estrogenic activities for the prenylated flavonoids examined. Furthermore, the activities were inhibited by an estrogen receptor antagonist, suggesting that the activities were likely being mediated by the estrogen receptors. However, there were differences in the activity, attributable to the hydroxyl group at position 4’, which is absent in kushenol A. While the estrogenic activity of kurarinone and sophoraflavanone G has been reported before, to the best of our knowledge, there are no such reports on kushenols A and I. Therefore, this study represents the first report of their estrogenic activity.

## 1. Introduction

*S. flavescens* is a perennial herb of the genus *Sophora* (Fabaceae), which includes approximately 52 species that are widely distributed in Asia, Oceania, and the Pacific Islands. In Japan, *S. flavescens* grows wild in the mountains and fields, mainly in Honshu, Shikoku, and Kyushu. The root of *S. flavescens*, also known as kurara in Japanese, has a strong bitter taste and has been used medicinally to treat fevers, dysentery, jaundice, vaginal itching with leukorrhagia, abscesses, carbuncles, enteritis, leukorrhea, pyogenic infections of the skin, scabies, swelling, and pain. Recent studies have shown that *S. flavescens* can regulate body functions and has anti-aging, antitumor, and immune-protective effects. Its pharmacological components, including alkaloids (e.g., matrine), saponins (e.g., sophoraflavosides), and flavonoids (e.g., kushenols), contribute to these effects [1,2].

Among flavonoid derivatives, prenylated flavonoids have been receiving great attention because of their beneficial effects [3,4,5,6,7]. The biosynthesis of prenylated flavonoids is mediated by flavonoid prenyltransferases specific to the positions of the flavonoid rings used for specific flavonoids, with dimethylallyl pyrophosphate serving as a prenyl donor, generating *O*- or *C*-prenylated products [5,6,7]. Prenylated sidechains have wide variations, including those with 3,3-dimethylallyl, geranyl, lavandulyl, and farnesyl groups. The prenylation of flavonoids increases lipophilicity, which promotes their affinity for the cell membrane and thereby enhances activities such as cytotoxic, antibacterial, anti-inflammatory, and estrogenic activities [4]. The estrogenic activity of prenylated flavonoids was summarized in several review articles [3,4,5,6,7]. Meanwhile, prenylated flavonoids in *S. flavescens* have also been investigated to explore their applications and clarify the molecular mechanisms underlying their beneficial effects [4,8]; however, their estrogen-related applications are limited because of the lack of information about the estrogenic components in the plant.

Food contains a wide variety of estrogenic constituents, such as lignans, terpenes, and flavonoids, and some of them are termed phytoestrogens. Phytoestrogens have been under focus because of their associated benefits, such as the chemoprevention of cancers and improvement of menopausal syndromes, osteoporosis, endometriosis, prostatic hyperplasia, polycystic ovary syndrome, and Alzheimer’s disease, which can be attributed to their estrogenic actions [9]. Estrogen is an important hormone because of its association with physiological and pharmacological effects, such as chromatin/epigenetic regulation, apoptosis, autophagy, cellular metabolism, translational control, cell cycle/DNA damage control, cytoskeletal/adhesion regulation, immunological/inflammatory response, neurodegenerative diseases, and development/differentiation, which are ascribed to its action at the molecular level [9,10]. Two major pathways, both genomic and non-genomic, are involved in the complex mechanism behind estrogen activity, initiated by its interaction with estrogen receptors (ERs), such as ERα, ERβ, and G-protein-coupled estrogen receptor 1 (GPER). These pathways are mediated by the complex signaling pathways, comprising the intracellular networks, in a process involving receptor crosstalk and signal bypass [10].

To evaluate the estrogenic activity of chemicals with diversified structures that act using such complex mechanisms, a wide variety of assays have been developed, such as ligand binding, yeast two-hybrid, reporter-genes, and transcription, protein, and cell assays, along with animal testing. These are based on the steps of estrogen action, ligand–receptor binding, co-regulator activation, promoter activation, mRNA production of marker/target genes (transcription), the production of marker/target proteins (translation), and the mortality/morphology/growth of live cells or organisms, respectively [10]. However, conflicting activities are often reported, such as estrogenic and anti-estrogenic activities for the same chemicals, owing to the differences in the assays and underlying mechanisms of estrogen action, as well as the additional activities associated with the chemicals. For example, cell and transcription assays are often used to evaluate the effect of chemicals after culturing cells for one or several days, while protein assays are based on the evaluation of the signaling for specific signal mediators within a few minutes to an hour, giving the information about different aspects of estrogenic actions. In this report, we utilized two mediators in estrogen signaling, namely extracellular signal-regulated kinases 1 and 2 (Erk1/2) and protein kinase B (Akt). These mediators are commonly employed to detect the rapid estrogen signaling induced by the membrane-bound ER, as reported in previous studies [11,12]. Thus, estrogenic activity has been evaluated by multiple assays, often including an analysis of the cell signaling pathway to help explain the activity at the molecular level [12,13].

**Figure 1:**
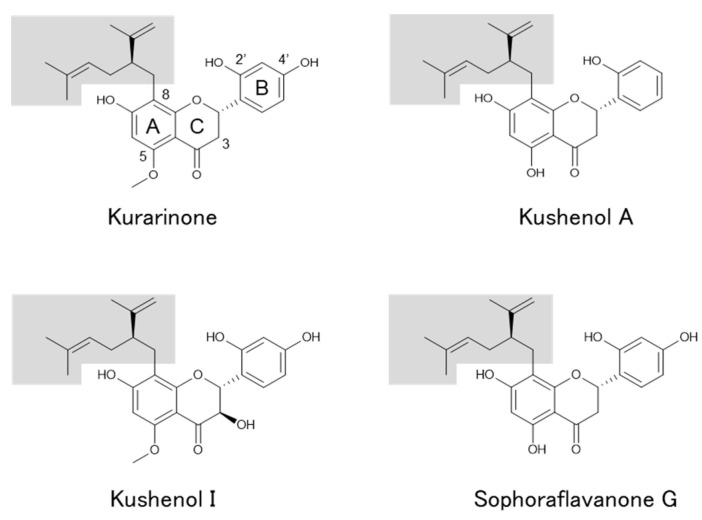
Structure of prenylated flavonoids in *S. flavescens*. The lavandulyl group is highlighted.

In this study, we use three assays to confirm the estrogenic activity of both kurarinone and sophoraflavanone G (Figure 1), which have been reported in previous studies. Both prenylated flavonoids have been found to be abundant in the root of *S*. *flavescens* [14,15]. Additionally, to the best of our knowledge, we present the first report on the estrogenic activity of kushenol A and I (Figure 1) [14], compounds with slightly different structures.

## 2. Materials and Methods

### 2.1. Materials

Kurarinone was purchased from Sigma-Aldrich (St. Louis, MO, USA), kushenol A from BioBioPha (Kunming, China), and kushenol I and sophoraflavanone G from MedChemExpress (Monmouth Junction, NJ, USA). Antibodies against total Erk1/2 (T-Erk1/2; catalogue No. #9102), phospho-Erk1/2 (P-Erk1/2; #9101), total Akt (T-Akt; #4691), and phospho-Akt (P-Akt; #4060) were obtained from Cell Signaling Technology (Ipswich, MA, USA). Additionally, 17β-estradiol (E_2_) and ICI 182,780 (ICI: an estrogen receptor antagonist) were purchased from Sigma-Aldrich (St. Louis, MO, USA), and we acquired dimethyl sulfoxide (DMSO) from FUJIFILM Wako Pure Chemical (Osaka, Japan). Human breast cancer MCF-7 cells were obtained from the Japanese Collection of Research Bioresources Cell Bank, and consistently cultured in an RPMI 1640 medium at 37 °C in 5% CO_2_. RPMI 1640 (phenol red-free) and RPMI 1640, containing 10% (*v*/*v*) dextran-coated charcoal-treated FBS (DCC-FBS), were purchased from Gibco, Thermo Fisher Scientific (Waltham, MA, USA).

### 2.2. Sulforhodamine B (SRB) Assay

The SRB assay was conducted following a protocol reported by Nishi et al. [12]. MCF-7 cells were cultured in phenol red-free RPMI 1640 medium supplemented with 10% fetal bovine serum. At the initial step of the SRB assay, the cells were seeded at an initial density of 1.5 × 10^4^ cells per well in a 24-well plate and incubated for 3 days in RPMI 1640 containing 10% (*v*/*v*) DCC-FBS. After 3 days, the medium was replaced by a new medium, where the cells were treated with 10 nM E_2_, indicated concentrations (10 nM, 100 nM, 1 μM, or 10 μM) of prenylated flavonoids (kurarinone, kushenols A or I, or sophoraflavanone G), 0.1% DMSO (vehicle control; Cont), or 1 μM ICI for 3 more days. All compounds were dissolved in serum-free RPMI 1640 before use. After treating the cells with chemicals, 200 µL of trichloroacetic acid (TCA; Sigma-Aldrich) was directly added to the 24-well plates, and the cells were fixed by placing them at conditions of 4 °C for 30 min. The fixed cells were washed with ultrapure water and precipitated protein was stained with 200 µL of 0.4% SRB (Sigma-Aldrich) in 1% acetic acid at room temperature for 20 min. The samples were again washed with 1% acetic acid and the stained protein was dissolved in 10 mM unbuffered Tris base (pH = 10.5) at room temperature for 10 min, and then placed in 96-well plates to measure absorbance at 490 nm using the SpectraMax ABS Plus (Molecular Devices; San Jose, CA, USA). The absorbances of DMSO and compound samples were compared for statistical analysis after subtracting the absorbance of 10 mM Tris as a background. Then, the ratio of the reads for the compounds to that for DMSO was calculated. After conducting the assay with three independent cell cultures, the mean and standard deviation (SD) were calculated and graphs were generated based on the results.

### 2.3. Western Blotting

Western blotting was also performed based on previously described methods [11,12]. First, MCF-7 cells were seeded in a 6-well plate at an initial density of 10^5^ cells per well and incubated for 2 days in RPMI 1640 containing 10% (*v*/*v*) DCC-FBS. Subsequently, all media in this 6-well plate were replaced by RPMI 1640 (serum-free) and incubated for 1 more day. Then, the cells were treated with 10 nM E_2_, 100 nM of prenylated flavonoids (kurarinone, kushenols A or I, or sophoraflavanone G), or vehicle (0.1% *v*/*v* DMSO) for 0, 5, 15, 30, and 60 min. If an inhibition experiment using ICI was performed, pre-treatment with ICI was applied for 1 h. After the treatment, all the media were discarded, and 200 µL of 1× sample buffer was added to each well to dissolve the cells. The lysates were sonicated for 30 s and finally heated at 95 °C for 5 min to prepare samples for Western blotting. Then, the protein in the lysates was separated through SDS-PAGE using e-PAGEL (ATTO; Tokyo, Japan) and was electro-blotted onto nitrocellulose membranes (Millipore; Billerica, MA, USA) using a semi-dry transfer cell (Bio-Rad Laboratories; Hercules, CA, USA). The membranes were first blocked with EzBlock BSA (ATTO) and then incubated with the antibodies against Erk1/2 or Akt after appropriate dilution (1:500 or 1:1000) using EzBlock BSA. The antigen–antibody complexes were further incubated with horseradish peroxidase (HRP)-coupled goat antibody against rabbit IgG (Cell Signaling Technology) after dilution (1:1000) with EzBlock BSA. The HRP signal was detected using WSE-6100 LuminoGraph I (ATTO) and WSE-7120 EzWestLumi plus (ATTO). The signals from total (T-) and phosphorylated (P-) proteins (Erk1/2 or Akt) were used for statistical analysis, and the P-protein/T-protein ratio was calculated. The experiment was independently performed three times, mean and SD were calculated, and the results were used for graph generation.

### 2.4. Real-Time Reverse Transcription-Polymerase Chain Reaction (RT-PCR)

The details of *real-time* RT-PCR have been previously described [12,13]. Briefly, MCF-7 cells were initially seeded at a density of 1.0 × 10^6^ cells per well in a 10 cm dish with RPMI 1640 containing 10% (*v*/*v*) DCC-FBS and incubated for 3 days. Then, the medium in the dishes was replaced with RPMI 1640 (serum-free) containing each compound, and stimulation was continued for additional 3 days. Total RNA was extracted from the cells using an RNeasy Mini-kit (QIAGEN; Venlo, The Netherlands) and used for RT-PCR reaction using an iTaq Universal SYBR Green One-Step kit (Bio-Rad Laboratories). *Real-time* RT-PCR was performed using the CFX Connect *real-time* PCR detection system (Bio-Rad Laboratories). RT-PCR consisted of the following steps. cDNA was synthesized at 42 °C for 10 min. Following denaturation at 95 °C for 1 min, PCR was carried out across 46 cycles, including denaturation at 94 °C for 10 s, annealing at 57 °C for 30 s, and extension at 72 °C for 20 s. The process was repeated three times using independent preparations of the cells treated with 10 nM E_2_, 100 nM, or 1 μM of the prenylated flavonoids (kurarinone, kushenols A or I, or sophoraflavanone G) or with vehicle (0.1% *v*/*v* DMSO). The ratios of the estimated amounts of mRNA for the compounds to that for the control (DMSO) sample were calculated for the respective genes using CFX Maestro Software (Bio-Rad Laboratories). The obtained values were normalized using the value for the β-actin gene and this was followed by log_2_-transformation. Subsequently, the values for a set of 30 estrogen-responsive genes [12] were evaluated by correlation analysis. The nucleotide sequences of PCR primers, except for *GATA4*, were as previously described [12], whereas the forward and reverse primers for *GATA4* were 5′-TCCAAACCAGAAAACGGAAG-3′ and 5′-CTGTGCCCGTAGTGAGATGA-3′, respectively.

### 2.5. Statistical Analysis

The mean ± SD values for all data were analyzed using Microsoft^®^ Excel (Microsoft; Seattle, WA, USA) and SPSS 12.0J (SPSS Japan; Tokyo, Japan). All statistical analyses were performed using a *t*-test and the significance was defined as *p* < 0.05 across all experimental sections. Subsequently, the correlation coefficient between gene expression profiles (*R*-value) was calculated using SPSS 12.0J based on linear regression.

## 3. Results

### 3.1. Evaluation of Estrogenic Activity by Cell Assay

**Figure 2:**
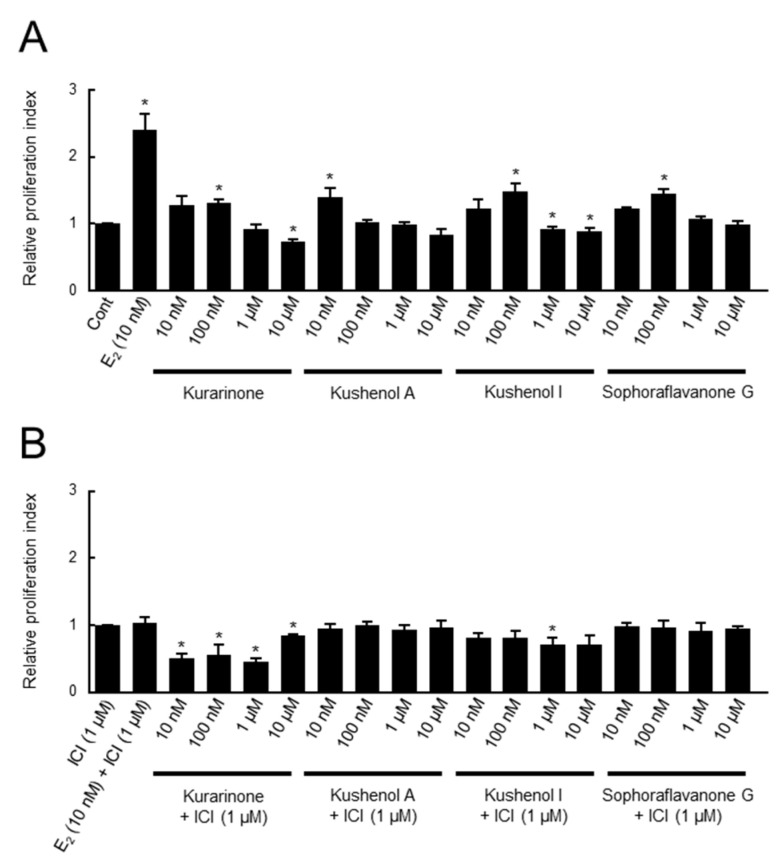
Evaluation of estrogenic activity of prenylated flavonoids via SRB assay. The estrogenic activity of the four prenylated flavonoids was evaluated by SRB assay. MCF-7 cells were treated with the indicated concentrations (10 nM to 10 μM) of kurarinone, kushenols A and I, and sophoraflavanone G in the absence (panel **A**) or presence (panel **B**) of ICI 182,780 (ICI) for 3 days, and the cell proliferation was evaluated via SRB assay. The relative proliferation indexes, indicating the degrees of cell proliferation for each treatment relative to those for vehicle treatment (0.1% DMSO; Cont) in panel **A** or vehicle treatment with 1 μM ICI in panel **B**, were calculated and are shown in the figure. Data from three independent experiments were statistically evaluated using a *t*-test: * *p* < 0.05. E_2_: 17β-estradiol. Y axis: cont. vs. each compound (**A**) or ICI vs. each compound + ICI (**B**).

We first examined the estrogenic activity of four prenylated flavonoids from *S. flavescens*, namely kurarinone, kushenols A and I, and sophoraflavanone G, by means of the cell proliferation assay using SRB (Figure 2). Among the concentrations examined (10 nM to 10 μM), kurarinone, kushenol I, and sophoraflavanone G showed the highest relative proliferation indexes at 100 nM, while kushenol A showed the highest value at 10 nM (Figure 2A). All the prenylated flavonoids exhibited inhibitory effects on cell proliferation at higher concentrations, suggesting the presence of cytotoxic activity. The cell proliferation activities of all the prenylated flavonoids were inhibited by the presence of the ER antagonist ICI (Figure 2B), suggesting that the activities were likely mediated by ERs.

### 3.2. Evaluation of Estrogenic Activity by Protein Assay

**Figure 3:**
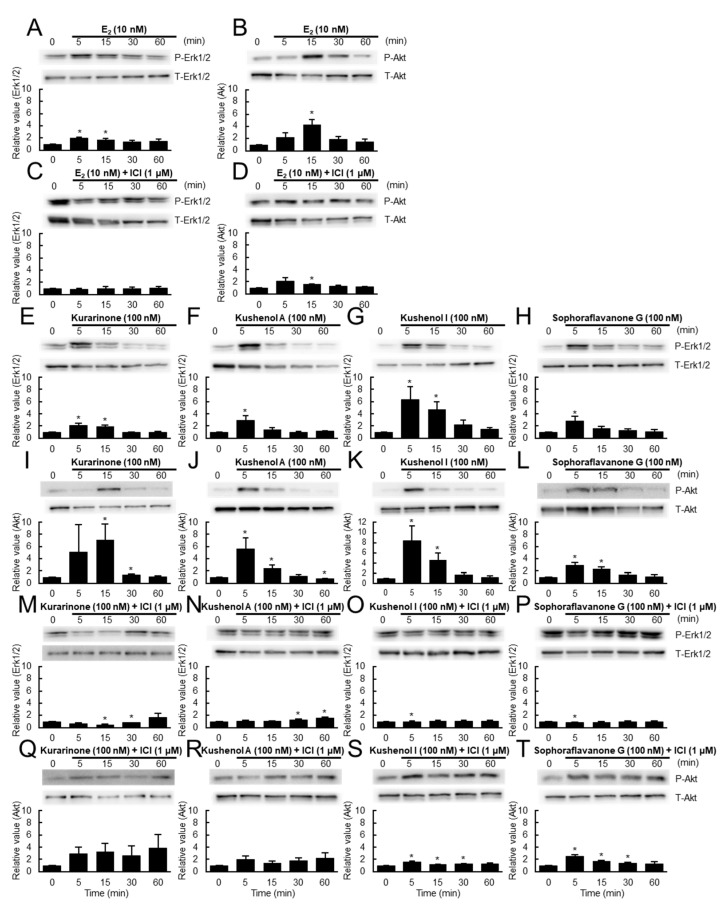
Evaluation of estrogenic activity of the four prenylated flavonoids by Western blotting. MCF-7 cells were treated with 10 nM E_2_ (panels (**A**–**D**)), 100 nM prenylated flavonoids (kurarinone, kushenols A or I, or sophoraflavanone G) (panels (**E**–**H**,**M**–**P**) for Erk1/2, and panels (**I**–**L**,**Q**–**T**) for Akt), or with vehicle (0.1% DMSO; 0 min) in the absence (panels (**A**,**B**,**E**–**L**)) or presence (panels (**C**,**D**,**M**–**T**)) of 1 μM ICI for the indicated times (min). The cell extracts were then subjected to Western blotting. The relative amounts of phosphorylated protein to that of total protein are shown in the graphs. Data from three independent experiments were statistically evaluated using a *t*-test; * *p* < 0.05. E_2_: 17β-estradiol.

We next examined the estrogenic activity of the four prenylated flavonoids via Western blotting (Figure 3). The estrogen signaling could be detected within 5 (for Erk1/2, Figure 3A) or 15 (for Akt, Figure 3B) min after the stimulation of MCF-7 cells with estrogen due to increased amounts of the phosphorylated forms of the mediators (P-Erk1/2 and P-Akt) over the amounts of the total proteins (T-Erk1/2 and T-Akt), which was detected by Western blotting. All the prenylated flavonoids at 100 nM exhibited increased amounts of P-Erk1/2 and P-Akt (Figure 3E–H for Erk1/2 and Figure 3I–L for Akt) within 5–15 min and the amounts returned to the control level at 60 min, suggesting that these prenylated flavonoids exhibited a quick estrogenic response. It is important to note that kushenol I exhibited a strong response. These responses were, however, inhibited by the presence of ICI (Figure 3C,D for E_2_ and Figure 3M–T for the prenylated flavonoids), suggesting that they were mediated by ERs.

### 3.3. Evaluation of Estrogenic Activity by Transcription Assay

**Figure 4:**
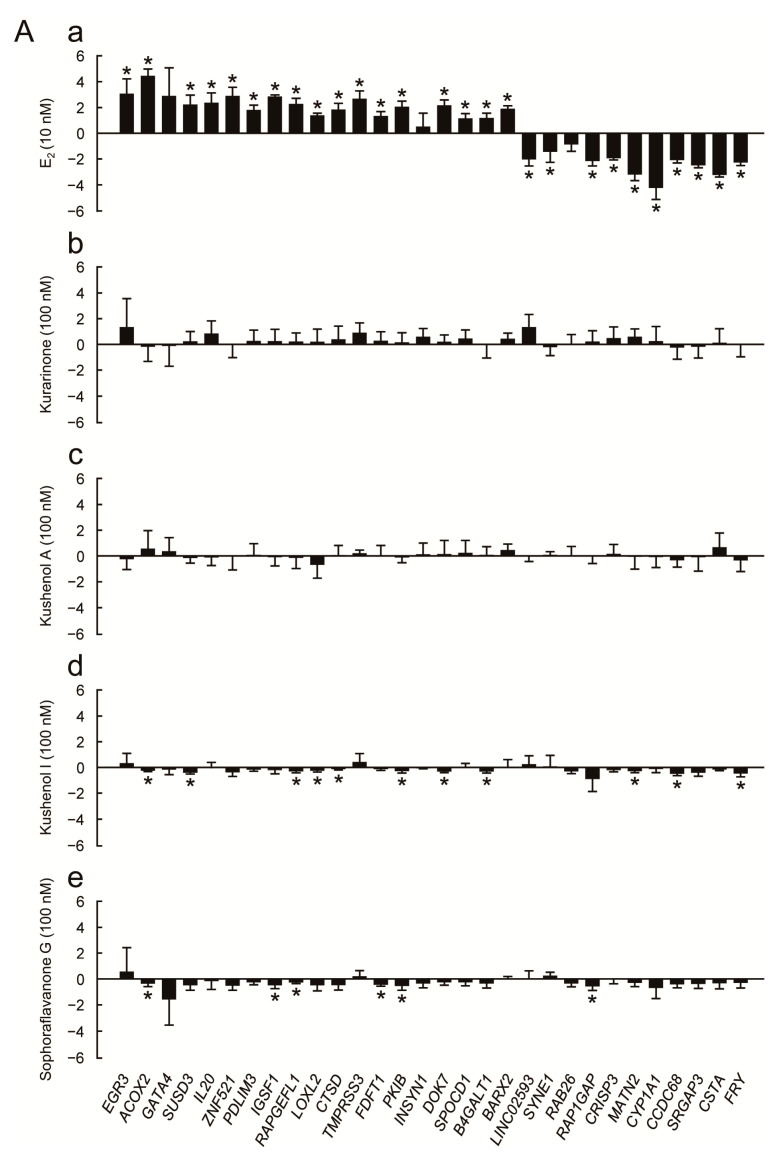

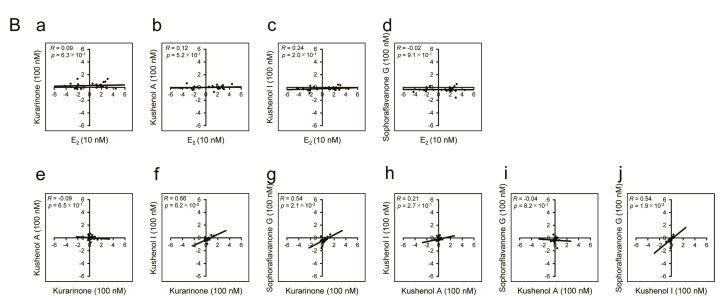
Evaluation of estrogenic activity of the four prenylated flavonoids (100 nM) by *real-time* RT-PCR. (**A**) The results of *real-time* RT-PCR using the designated 30 estrogen-responsive genes for E_2_ (**a**), kurarinone (**b**), kushenol A (**c**), kushenol I (**d**), and sophoraflavanone G (**e**). The graphs show the expression level of each estrogen-responsive gene (the name is shown at the bottom), obtained via *real-time* RT-PCR, and the bars show the mean ± SD (n = 3) of log_2_-transformed ratios of the data obtained in the presence of each chemical to those of data acquired in their absence (E_2_+/E_2_−, for example) in the Y axis. * *p* < 0.05: between each compound and the control (vehicle; 0.1% DMSO). (**B**) Correlation analysis of the profiles obtained by *real-time* RT-PCR. The gene expression profiles for the set of 30 estrogen-responsive genes were compared between E_2_ and the four prenylated flavonoids (panels **a**–**d**), or within the four prenylated flavonoids (panels **e**–**j**), based on linear regression, and the results were visualized as scatter plot graphs. The vertical and horizontal axes indicate log_2_ values for the indicated chemicals. The correlation coefficients (*R*-values) and the values for the significance of correlation (*p*-values) are indicated. E_2_: 17β-estradiol.

**Figure 5:**
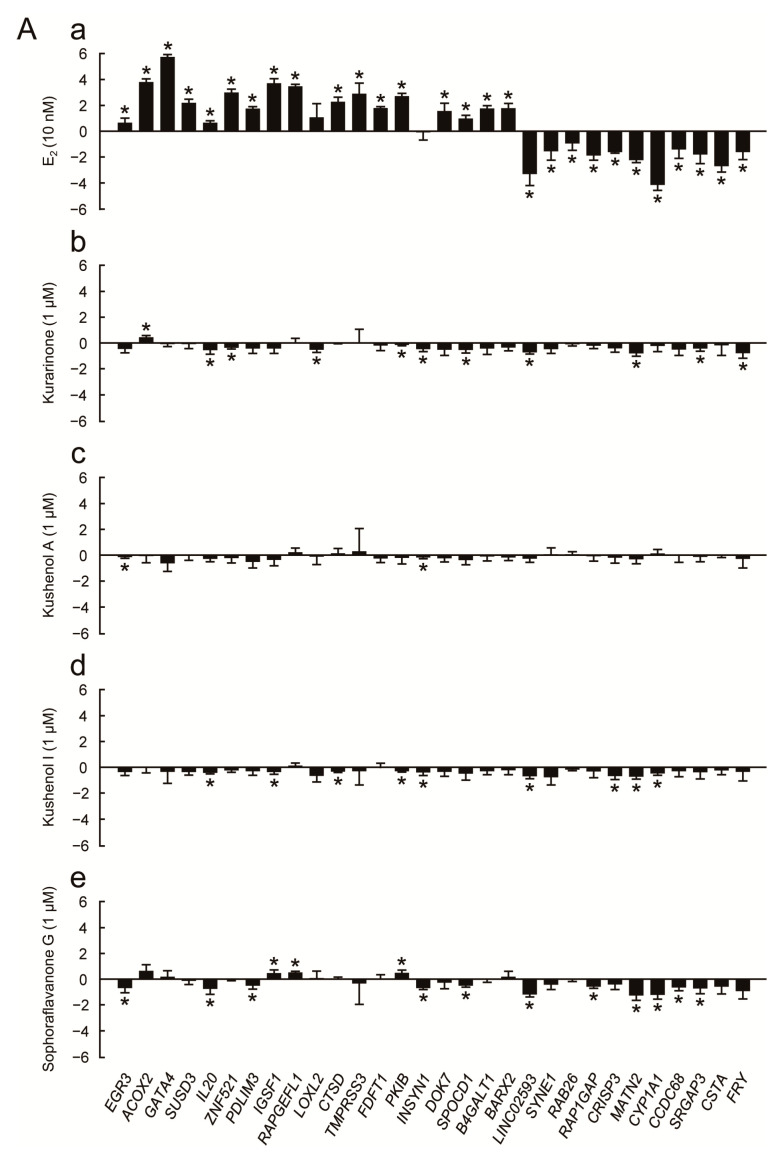

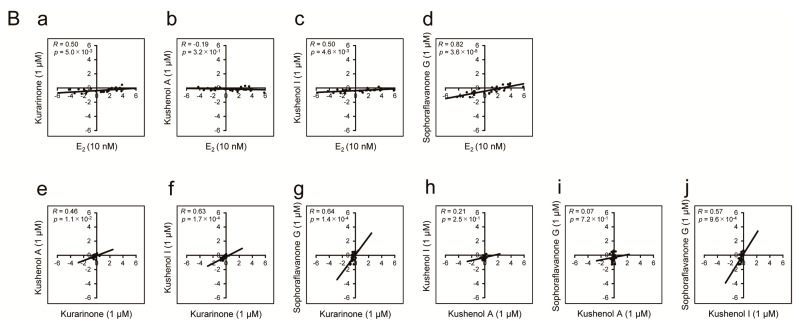
Evaluation of estrogenic activity of the four prenylated flavonoids (1 μM) by *real-time* RT-PCR. (**A**) The results of *real-time* RT-PCR (n = 3) for each of the designated 30 estrogen-responsive genes for E_2_ (**a**), kurarinone (**b**), kushenol A (**c**), kushenol I (**d**), and sophoraflavanone G (**e**). For details, see Figure 4 legend. (**B**) Correlation analysis of the profiles obtained by *real-time* RT-PCR. The gene expression profiles for the set of 30 estrogen-responsive genes were compared between E_2_ and the four prenylated flavonoids (panels **a**–**d**), or within the four prenylated flavonoids (panels **e**–**j**), based on linear regression, and the results were visualized as scatter plot graphs. The vertical and horizontal axes indicate log_2_ values for the indicated chemicals. The correlation coefficients (*R*-values) and the values for the significance of correlation (*p*-values) are indicated. E_2_: 17β-estradiol.

We then conducted transcription assays to examine the estrogenic activity of the four prenylated flavonoids (Figure 4 and Figure 5). We used the 30 estrogen-responsive genes that were selected via RNA sequencing based on their reliable response to estrogen [12]. These genes are associated with various cell functions, such as those involving enzymes (metabolic enzymes, proteases, and protein kinases), cell signaling mediators, tumor suppressors, and transcriptional regulators, as well as various cell signaling pathways, including estrogen signaling [12]. The expression profiles of each of the 30 estrogen-responsive genes in the presence and absence of chemicals are shown in Figure 4A and Figure 5A, followed by the results of a correlation analysis of the chemicals (Figure 4B and Figure 5B). We examined the profiles at two different concentrations (100 nM in Figure 4 and 1 μM in Figure 5) for the treatment of the prenylated flavonoids because the prenylated flavonoids exhibited optimal activities at different concentrations (see Figure 2A). While the prenylated flavonoids responded to MCF-7 cells in the cell assay (Figure 2A), their responses in the transcription assay were weak (Figure 4A and Figure 5A). Furthermore, the gene expression profiles of the prenylated flavonoids exhibited very low or no correlation with those of E_2_ at 100 nM concentrations (Figure 4B), and low to medium levels of correlation at 1 μM (Figure 5B). However, based on the pairwise comparisons among the prenylated flavonoids (panels e to j in both Figure 4B and Figure 5B), the profiles exhibited significant levels of correlation (*R* > 0.5), except for kushenol A (panels e, h, and i in both Figure 4B and Figure 5B). Kushenol A exhibited low levels of correlation, although there were some correlations at 1 μM (Figure 5B). This difference was also observed in the cell assay (Figure 2), and this was potentially due to the structural differences among the four prenylated flavonoids, such as the presence (kurarinone, kushenol I and sophoraflavanone G) and absence (kushenol A) of the hydroxyl group at position 4’ (see Section 4: Discussion).

## 4. Discussion

### 4.1. Estrogenic Response Induced by Prenylated Flavonoids in S. flavescens

Among the prenylated flavonoids reported in *S. flavescens*, we examined four, namely kurarinone, kushenols A and I, and sophoraflavanone G, and determined whether they exhibit estrogenic activity. All of them contain a *C*-linked lavandulyl group at position 8 of ring A, but they have differences in the hydroxyl group at positions 3 (ring C), 5 (ring A), and 4’ (ring B), giving rise to similar but distinctive structural and functional characteristics. While the estrogenic activity of kurarinone and sophoraflavanone G have been reported (see the later discussion on estrogenic flavonoids), to the best of our knowledge, there have been no such reports for kushenols A and I. Thus, this study may be the first to report their estrogenic activity.

There are two important structural aspects for chemicals, especially for phenolics such as alkylphenols, in terms of exerting estrogenic activity. First, there is a polycyclic planar ring system mimicking the steroid AB-ring, important for binding with ERs [16]. Second, there is the substitution of the sidechains of alkylphenols, contributing to the strength of their estrogenic activity [17]. In particular, alkylphenols substituted at position 4 with 6- to 8-carbon-containing, tertiary alkyl groups exhibit higher activities. As for 17β-estradiol, the hydroxyl groups at positions 3 (ring A) and 17 (ring D), as well as the hydrophobicity of its rings B and C, are important structural features [18]. Thus, prenylation (lavandulylation) and hydroxyl group substitutions may contribute to the estrogenic activity of the four prenylated flavonoids examined here (see the later discussion about estrogenic activity of prenylated flavonoids).

**Figure 6:**
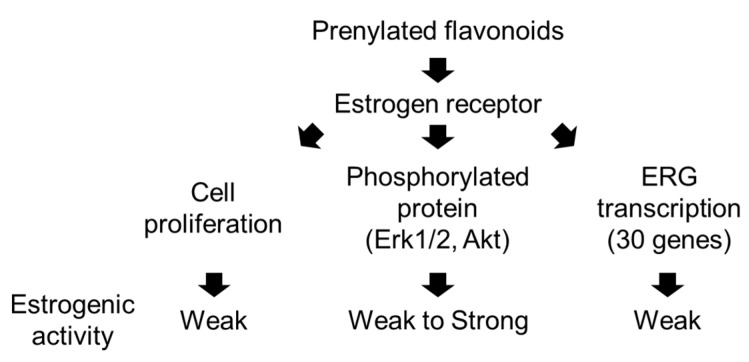
Summary of results.

Here, we employed three assays to evaluate estrogenic activity (summarized in Figure 6), namely the cell proliferation assay using SRB (Figure 2), Western blotting (Figure 3), and *real-time* RT-PCR (Figure 4). These corresponded to cell, protein, and transcription assays, respectively, as classified by the molecular and cellular mechanisms of estrogen action [10]. All the assays provided important information regarding the mechanisms of estrogen action. The cell assay was based on the detection of cell growth and proliferation by evaluating cell size, shape, viability, type, and functional states such as cell cycle, apoptosis, and migration.

The protein assay has been used to quantitatively detect protein, where one or several protein markers are detected using radio-, enzyme-, and fluoro-based assays or through physicochemical methods, such as mass spectrometry. We used Western blotting to detect two types of signaling proteins, Erk1/2 and Akt, as markers for estrogenic activity [11,12]. Estrogens and estrogenic chemicals activate the pathways involving these kinases by binding with ERs, including GPER, to exert cellular functions such as cell proliferation. Finally, the transcription assay is based on the analysis of gene transcripts, where the expression of ER genes, specific marker genes, or estrogen-responsive genes can be quantified to evaluate responsiveness to estrogen stimulation.

We used a set of 30 estrogen-responsive genes that provided a stable, reliable, and quantitative evaluation of estrogenic activity [12,13]. All the assays indicated weak but clear estrogenic activities for the prenylated flavonoids examined. Furthermore, the activities were inhibited by an ER antagonist, ICI, suggesting that they were likely mediated by ERs. Based on the results of the estrogen assays employed, especially those of cell and protein assays (Figure 2 and Figure 3), we concluded that the four prenylated flavonoids, kurarinone, kushenols A and I, and sophoraflavanone G, exerted estrogenic activity through both the genomic and non-genomic pathways. Furthermore, the rapid estrogenic responses observed in the protein assay (Figure 3) suggested the involvement of membrane ERs. On the other hand, the results of the transcription assay (Figure 4 and Figure 5) indicated that, although the profiles of the four prenylated flavonoids were different from that for E_2_, there were clear similarities among them (see Section 3: Results). This suggested the presence of similar mechanisms for the responses at the transcription level. Furthermore, the differences in profiles between E_2_ and the four prenylated flavonoids (panels a to d in both Figure 4B and Figure 5B) indicated differences in the mechanisms of estrogenic response as a whole. It should be noted that the differences among the four prenylated flavonoids in cell and transcription assays (Figure 2, Figure 4 and Figure 5) were likely due to the presence and absence of the hydroxyl group at position 4’ in ring B. It is important to note that the hydroxyl group at position 4’ was suggested as contributing to estrogenic activity (reviewed in Kiyama, 2023 [19]). However, it should be noted that many of the 30 estrogen-responsive genes responded to the prenylated flavonoids, suggesting that the observed responses were estrogenic. We also examined the estrogenic activity of other flavonoids in *S. flavescens* based on reference searches (see Section 4.2).

Based on the results of correlation analysis (Figure 4 and Figure 5) and cluster analysis (Figure S1), sophoraflavanone G showed a high correlation with E_2_. In contrast, kushenol I and kurarinone exhibited similar correlation values compared to E_2_, while kushenol A demonstrated the lowest value among the four prenylated flavonoids. This difference may be attributable to the number of hydroxyl groups and their positions. Sophoraflavanone G and kushenol I, respectively, have a total of four hydroxyl groups, whereas the others have three. The results suggested that the positioning of the hydroxyl group at position 4’ in ring B potentially contributed greatly to the estrogenic activity because the lack of it in kushenol A may have resulted in a low estrogenic activity. In contrast, the hydroxyl group at position 3 in ring C may not have significantly influenced the results of the transcription assay, as evidenced by the similar correlation values for kurarinone and kushenol I. Therefore, both the number and location of hydroxyl groups may be crucial factors influencing estrogenic activity.

### 4.2. Estrogenic Flavonoids in S. flavescens

Among the 124 flavonoids isolated from the roots of *S. flavescens* [1], 17 chemicals, including those investigated here, were reported to exhibit estrogenic activity ([19]; summarized in Table 1). These flavonoids exhibited either estrogenic (including agonistic) or anti-estrogenic activity, or both, along with other characteristics such as biphasic actions (estrogenic at low concentrations and anti-estrogenic at high concentrations) and acting as a selective estrogen receptor modulator (SERM). Among them, chalcones (xanthohumol), flavanones/dihydroflavonols (isoxanthohumol, kurarinone, kushenols A, F, I and X, leachianone A, and sophoraflavanone G), and a flavonol (kushenol C) belonged to prenylated flavonoids (Table 1). The prenylation of flavonoids occurs in chalcones/dihydrochalcones, flavanones, flavones, flavonols, and isoflavones in the form of *C*- or *O*-prenylation, giving rise to sidechains such as 3,3-dimethylallyl, 1,1-dimethylallyl, geranyl, lavandulyl, and farnesyl groups, along with further modifications through oxidation, reduction, dehydration, and cyclization [20]. Compared to their parent compounds, prenylated flavonoids have certain advantages, including greater inhibitory effects on enzymes related to cell signaling and biological activities, increased benefits regarding bone and muscle maintenance, improved antioxidant activity for immune response, enhanced antitumor activity due to cytotoxic effects, and stronger estrogenic activity for endocrine functions [4,21].

Prenylation may improve the affinity of a phytoestrogen for ERs and the selectivity between ERs, although this depends on the position of the modification [5]. The contribution of the prenyl group to the increase in estrogenic activity was demonstrated by comparing the activity between naringenin and its prenylated products, 6- and 8-prenylnaringenins [22]. Furthermore, prenylation of flavonoids at C8 with C2–C3 unsaturation increases ERβ selectivity [23]. The estrogenic activity of prenylated flavonoids, including the ones listed in Table 1, has been summarized in several review articles [7,19]. For example, the effects of xanthohumol, a prenylated chalcone, on the suppression of ERs and inhibition of breast cancer development have been reported [24]. Although 8-prenylnaringenin exhibits strong estrogenic activity, its metabolic precursors, xanthohumol (a precursor of cyclization) and isoxanthohumol (a precursor of *O*-demethylation), show weak or no estrogenic activity [19,25,26]. This suggests that, while prenyl sidechains contribute to estrogenic activity, other parts also play roles.

**Table 1:** Estrogenic flavonoids in *S. flavescens*.

Chemical	Flavonoid Class	Estrogenicity ^a^	Reference
**Chalcone and Dihydrochalcone**			
Xanthohumol	Chalcone (prenylated)	A	Yoshimaru et al., 2014 [24]
**Flavanone and Dihydroflavonol**			
Isoxanthohumol	Flavanone (prenylated)	E	Żołnierczyk et al., 2015 [26]
Kurarinone	Flavanone (prenylated)	E	De Naeyer et al., 2004 [27];
			Wang et al., 2011 [28]; This study
Kushenol A	Flavanone (prenylated)	E	This study
Kushenol F	Flavanone (prenylated)	E	Wang et al., 2011 [28]
Kushenol I	Dihydroflavonol (prenylated)	E	This study
Kushenol X	Dihydroflavonol (prenylated)	L	Hillerns and Wink, 2005 [29]
Leachianone A	Flavanone (prenylated)	L	Hillerns and Wink, 2005 [29]
Naringenin	Flavanone	E/A/S/L/B	Kiyama, 2023 [19]
Sophoraflavanone G	Flavanone (prenylated)	L	Hillerns and Wink, 2005 [29];
			This study
**Flavone and Flavonol**			
Kushenol C	Flavonol (prenylated)	L	Hillerns and Wink, 2005 [29]
Quercetin	Flavonol	E/A/B	Kiyama, 2023 [19]
Rutin	Flavonol (glycoside)	E	Kiyama, 2023 [19]
**Isoflavone**			
Biochanin A	Isoflavone	E/A/S/B	Kiyama, 2023 [19]
Calycosin	Isoflavone	E/A/S/B	Kiyama, 2023 [19]
Daidzein	Isoflavone	E/A/S	Kiyama, 2023 [19]
Formononetin	Isoflavone	E/A	Kiyama, 2023 [19]

Flavonoids in *S. flavescens*, listed in He et al. (2015) [1], were examined for estrogenicity. For the indicated flavonoids, see the complete list of references in the paper by Kiyama [19]. After the literature search, no reports were found for the following classes of flavonoids: anthocyanidin/anthocyanin, 2-arylbenzofuran/3-arylcoumarin/α-methyldeoxybenzoin, aurone, coumaronochromone, coumestan, flavan/flavan-3-ol/flavan-4-ol, homoisoflavonoid, isoflavan, isoflavanone, isoflavene, neoflavonoid, oligoflavonoid, pterocarpan/pterocarpene, or rotenone. ^a^ Estrogenicity: anti-estrogenic (A), biphasic (B), estrogenic (E), binding as a ligand (L), or SERM (S) (See paper by Kiyama [19] for details on estrogenic activity).

In a previous study, kurarinone (prenylated flavanone) showed weak estrogenic activity, with EC_50_ values of 1.66 in a yeast reporter gene assay and 4.6 µM in an alkaline phosphatase assay [27]. Similarly, kurarinone and kushenol F were shown to promote ER-dependent osteogenic differentiation and the mineralization of osteogenic cells [28]. Meanwhile, in another report, kushenol X, leachianone A and sophoraflavanone G (norkurarinone; prenylated flavanone or dihydroflavonol), and kushenol C (prenylated flavonol) exhibited weak binding to rat uterine ER [29]. In this report, the flavonoids with a lavandulyl group exhibited almost equivalent estrogenic activity to those with a prenyl group. Although sophoraflavanone G (8-lavandulyl-2′-hydroxynaringenin) can be produced biosynthetically through 8-lavandulylation and 2′-hydroxylation from 8-prenylnaringenin [30], which has strong estrogenic activity, it (norkurarinone) binds weakly to rat uterine ER (EC_50_ of 8.224 ± 4.138 μg/mL [29]). This was also observed in the present study.

### 4.3. Applications of Prenylated Flavonoids in S. flavescens

Several applications of prenylated flavonoids have been suggested, including cancer prevention [7] and other uses [31], which are based on their unique bioavailability and tissue-specific bioaccumulation [32]. Applications of the prenylated flavonoids found in *S. flavescens* have also been suggested, although few are related to estrogenic activity. For example, kurarinone has been reported to have anticancer potential against cervical, lung, hepatic, esophageal, breast, gastric, cervical, and prostate cancers, potentially by inhibiting cell cycle progression, inducing apoptosis, and suppressing metastasis of cancer cells through the modulation of signal mediators such as NF-κB, caspases, and cadherins [33]. Kurarinone also possesses anti-inflammatory, anti-drug resistant, anti-microbial, channel and transporter modulatory, neuroprotective, and estrogenic activities [33]. Meanwhile, sophoraflavanone G has antitumor, anti-inflammatory, antibacterial, and insecticidal activities, as well as inhibitory activities against phospholipase Cγ1, β-secretase, neuraminidase, tyrosinase, glycosidase, and Na^+^-glucose cotransporter [1], although its estrogen-related applications have not been reported. Applications of kushenols A and I have also been reported, including their use as enzyme inhibitors [34,35,36], anticancer agents [37,38], and in traditional Chinese medicine [39,40,41]. However, there have been no reports concerning their estrogenic activity, and thus, none on their estrogen-related applications [19].

## 5. Conclusions

This study, to the best of our knowledge, is the first to report the estrogenic activity of kushenol A and kushenol I, elucidating the underlying mechanisms through cell, protein, and transcription assays. While kushenol A did not exhibit sufficient activity for in-depth investigation, the various outcomes provide a basic understanding of the estrogenic activity of kurarinone, kushenols A and I, and sophoraflavanone G, especially regarding their structure–activity relationships. Our study’s findings may be utilized in future studies.

## Data Availability

The authors confirm that the data underlying this article are available in the article.

## Supplementary Materials

The following supporting information can be downloaded at: https://www.mdpi.com/article/10.3390/genes15020204/s1, Figure S1: Cluster analysis for E_2_ and the prenylated flavonoids.
