# Supplementary material for: Estrogenic Prenylated Flavonoids in Sophora flavescens
Source: Genes (Basel). 2024 Feb 4;15(2):204. doi: 10.3390/genes15020204 (PMC10887985; doi:10.3390/genes15020204)
Supplement: Supplementary file 1 [file genes-15-00204-s001.zip › genes-2777500-supplementary.pdf]

[Supplementary Materials]

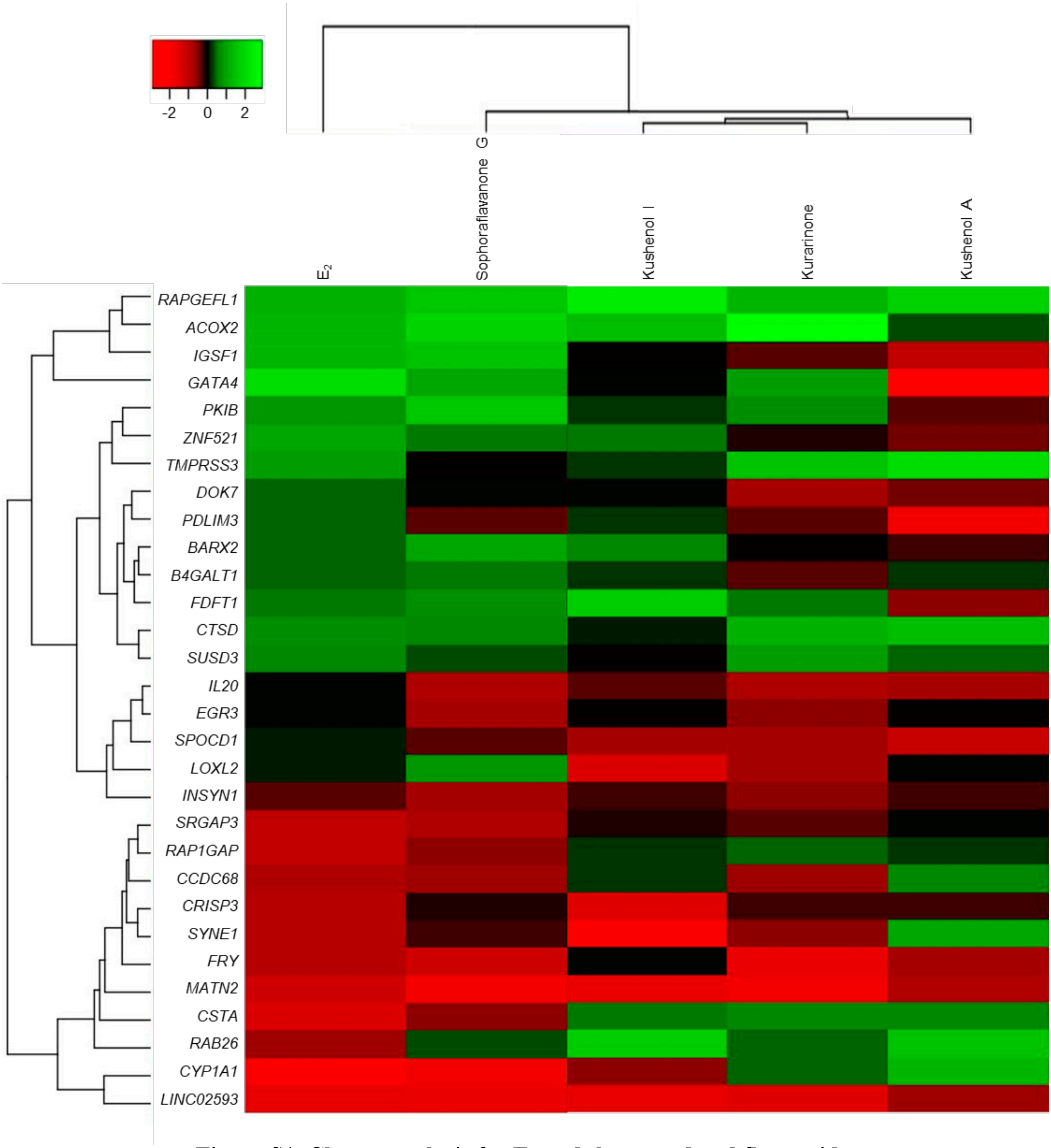

Figure S1. Cluster analysis for E_2 and the prenylated flavonoids.

The expression profiles of the 30 estrogen-responsive genes that were obtained for E_2 and the four prenylated flavonoids by *real-time* RT-PCR were analyzed by hierarchical clustering using heatmapper software (http://heatmapper.ca/expression).
